# Clinical Application of 3D‐Printed Custom Hemipelvic Prostheses With Re‐Entrant Chiral Structure in Reconstruction After Pelvic Tumor Resection

**DOI:** 10.1111/os.70159

**Published:** 2025-08-21

**Authors:** Linyun Tan, Ye Li, Xin Hu, Yitian Wang, Xiaolu Zhang, Xiaoyan Liu, Yi Luo, Yong Zhou, Chongqi Tu, Xiao Yang, Li Min

**Affiliations:** ^1^ Department of Orthopedic Surgery and Orthopedic Research Institute West China Hospital, Sichuan University Chengdu Sichuan China; ^2^ Model Worker and Craftsman Talent Innovation Workshop of Sichuan Province Chengdu Sichuan China; ^3^ Department of Orthopedics Surgery West China Hospital, Sichuan University/West China School of Nursing Chengdu Sichuan China; ^4^ National Engineering Research Center for Biomaterials, Sichuan University Chengdu China; ^5^ Provincial Engineering Research Center for Biomaterials Genome of Sichuan, Sichuan University Chengdu China

**Keywords:** 3D‐printed prosthesis, early clinical outcomes, negative Poisson's ratio, pelvic reconstruction, re‐entrant chiral structure

## Abstract

**Objectives:**

Pelvic reconstruction with conventional 3D‐printed prostheses faces a critical trade‐off, where achieving sufficient porosity for optimal bone ingrowth often compromises essential mechanical stability. To address this challenge, this study evaluates the clinical outcomes of 3D‐printed hemipelvic prostheses incorporating re‐entrant chiral structure (RCS), a novel negative Poisson's ratio design, in patients undergoing pelvic reconstruction following tumor resection.

**Methods:**

A retrospective analysis was conducted on 15 patients (eight females and seven males; mean age: 39.3 ± 11.7 years) with pelvic malignancies who underwent reconstruction using 3D‐printed hemipelvic prostheses incorporating RCS between March 2018 and June 2023. The diagnoses included osteosarcoma (*n* = 8), Ewing's sarcoma (*n* = 3), chondrosarcoma (*n* = 2), and high‐grade soft tissue sarcoma (*n* = 2). All patients were staged as IIB according to the Enneking system, except for one case of Ewing's sarcoma (stage III). Neoadjuvant chemotherapy (four cycles) was administered to six osteosarcoma patients, and one Ewing's sarcoma patient received six cycles, while other patients proceeded directly to surgery. Patient outcomes were systematically evaluated through oncological status, functional performance (MSTS‐93 score), pain assessment (VAS score), surgical parameters, complications, and radiographic analysis using Tomosynthesis Shimadzu Metal Artifact Reduction Technology (T‐SMART).

**Results:**

At the latest follow‐up (44.5 ± 9.4 months), 13 patients (86.7%) remained disease‐free; one patient (6.7%) experienced local recurrence requiring revision surgery, and one patient (6.7%) died of metastatic complications at 32 months post‐surgery. Functional outcomes showed significant improvement, with mean MSTS‐93 scores increasing from 14.5 ± 1.1 preoperatively to 25.8 ± 1.3 at final follow‐up (*p* < 0.001). Pain control was satisfactory, with VAS scores decreasing from 5.5 ± 0.6 to 1.5 ± 0.5 (*p* < 0.001). The mean surgical duration was 289.3 ± 30.4 min, with an average intraoperative blood loss of 3540 ± 621.5 mL. Early complications included delayed wound healing in three cases (20%), successfully managed with wound care protocols and VAC therapy. One patient (6.7%) developed deep prosthetic infection at 14 months post‐surgery, necessitating a two‐stage revision procedure. No mechanical failures, aseptic loosening, or prosthesis fractures were observed during the follow‐up period. Radiographic analysis demonstrated progressive bone ingrowth into the RCS porous regions in all cases, with no signs of osteolysis or implant migration in the remaining prostheses.

**Conclusion:**

D‐printed custom hemipelvic prostheses with RCS offer an effective solution for pelvic reconstruction by achieving an optimal balance between mechanical stability and biological integration, leading to promising clinical outcomes.

## Graphical abstract

Novel 3D‐printed hemipelvic prostheses incorporating re‐entrant chiral structure achieved 75% porosity while maintaining structural integrity. Clinical outcomes demonstrated 86.7% disease‐free survival and significant functional improvement (MSTS‐93: 14.4→25.7) in pelvic malignancy reconstruction.

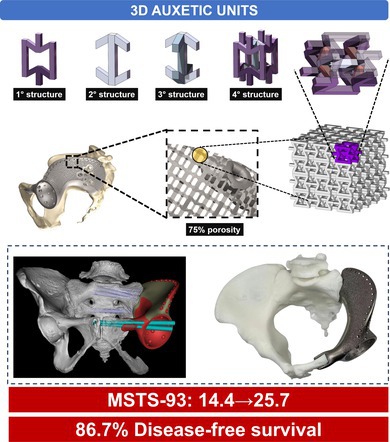

## 1 Introduction

Malignant pelvic tumors present significant challenges in orthopedic oncology, accounting for 5%–7% of primary bone malignancies [1]. While advances in surgical techniques and multimodal treatments have established limb salvage as the standard of care, pelvic reconstruction following tumor resection remains one of the most challenging procedures in orthopedic surgery [2, 3]. The complex anatomical architecture, substantial mechanical loads, and extensive defects following tumor resection create unique surgical challenges. Current reconstructive options include arthrodesis, transposition procedures, biological reconstructions with auto/allografts, and prosthetic replacements [4, 5, 6, 7, 8]. Among these, prosthetic reconstruction has emerged as a preferred approach, offering immediate stability and improved functional outcomes [9, 10, 11, 12, 13, 14, 15].

While titanium alloy prostheses have been widely adopted for their excellent mechanical properties and biocompatibility, the fundamental challenge of achieving optimal host‐implant integration persists. The emergence of 3D printing technology has revolutionized prosthetic design by enabling the creation of complex porous architectures that better mimic natural bone structure [16, 17, 18]. This advancement allows for precise control over pore size, distribution, and interconnectivity—critical factors for cellular infiltration and neovascularization. However, a significant limitation exists in current metallic porous implants: the need to maintain structural integrity necessitates restricting porosity to below 40%, which falls considerably short of the optimal parameters for osteoconductivity (porosity > 60%, pore size > 300 μm) [19, 20]. This constraint creates a critical trade‐off between mechanical strength and biological performance. Higher porosity would theoretically enhance bone ingrowth and reduce stress shielding by better matching the elastic modulus of natural bone, but traditional porous designs cannot achieve this without compromising structural stability [21]. Furthermore, conventional porous structures often exhibit inconsistent mechanical properties across different loading directions, potentially leading to localized stress concentrations and implant failure [22].

Recent advances in materials science have highlighted the potential of auxetic materials—structures with negative Poisson's ratio—to address this challenge. These materials exhibit the unique property of lateral expansion under tensile loading, conferring enhanced mechanical characteristics including superior impact resistance, fracture toughness, and energy absorption [23, 24]. In orthopedic applications, negative Poisson's ratio structures have shown promising results across various subspecialties. For instance, honeycomb‐patterned auxetic structures in hip prostheses have demonstrated improved bone–implant interface characteristics [25], while auxetic porous screws have shown enhanced fixation stability and reduced loosening rates in trauma applications [26, 27, 28, 29]. Building on these developments, we propose integrating RCS, a specialized auxetic architecture, into 3D‐printed hemipelvic prostheses.

Building on these advances in auxetic materials, this study introduces a novel hemipelvic prosthesis integrating re‐entrant chiral structure (RCS) architecture. We aim to evaluate its clinical performance by investigating three primary questions that link its theoretical biomechanical advantages to real‐world patient outcomes: (1) Clinical stability and durability: Given that the RCS architecture is designed for superior fatigue resistance and stress distribution, does this theoretical biomechanical advantage translate into a low rate of mechanical complications (e.g., aseptic loosening, prosthesis fracture, or structural failure) in the demanding, high‐load environment of the pelvis? (2) Effective biological integration: Does the combination of high porosity (> 60%) and a bone‐matched mechanical environment within the RCS prosthesis promote reliable and progressive osseointegration at the bone–implant interface, as evidenced by serial radiographic analysis? (3) Overall clinico‐functional efficacy: Ultimately, do these favorable biomechanical and biological attributes culminate in tangible patient benefits, specifically significant functional recovery, effective pain management, and satisfactory oncological control following major pelvic tumor resection?

This study presents the clinical outcomes of the first 15 consecutive patients treated with these RCS‐enhanced prostheses, aiming to provide initial evidence for their safety and efficacy in this challenging application.

## 2 Methods

### 2.1 Development of Novel Three‐Dimensional Re‐Entrant Chiral Architecture

**FIGURE 1:**
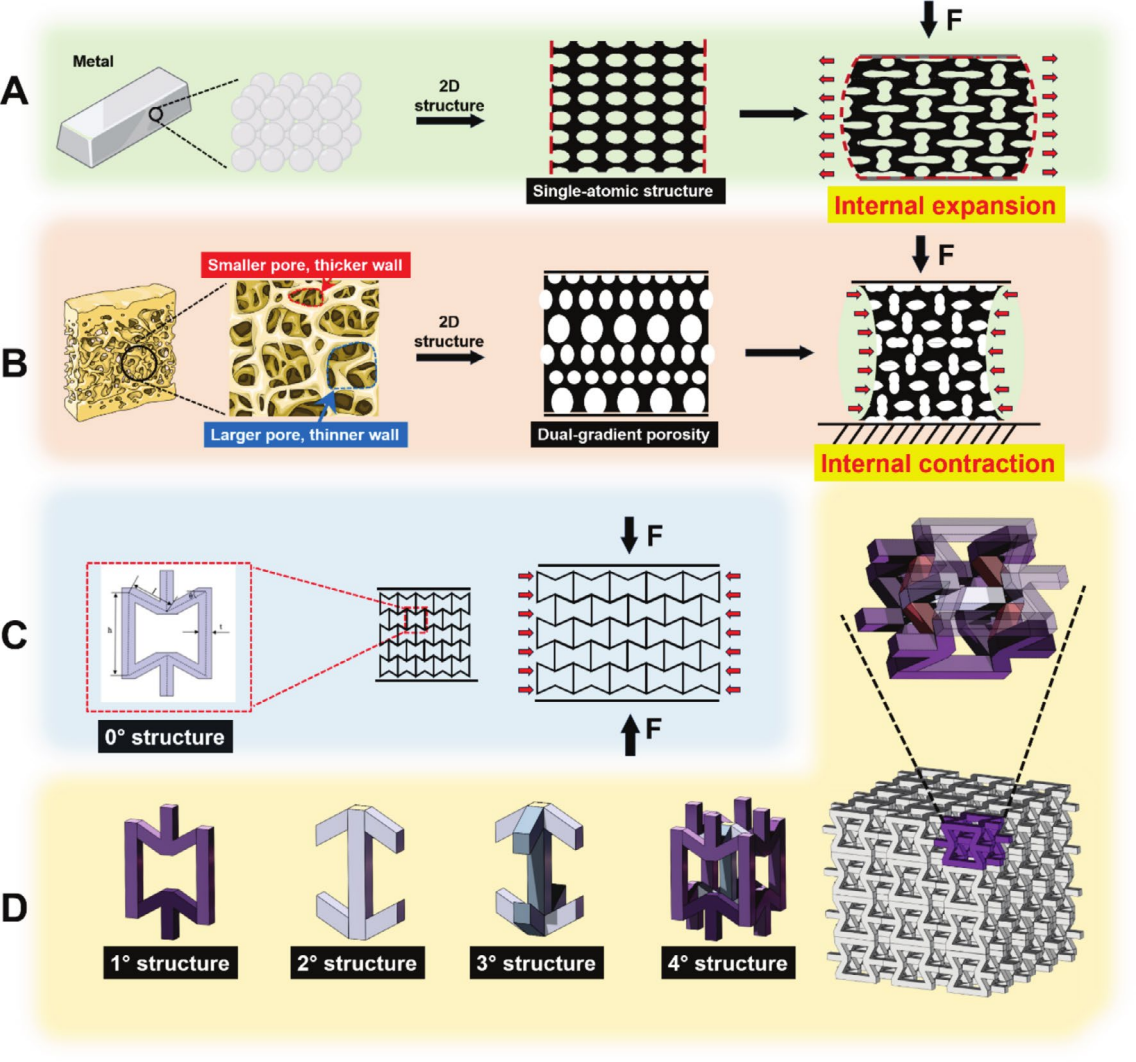
Design of three‐dimensional RCS units. (A) The metallic structure with positive Poisson's ratio exhibits internal expansion characteristics under axial compression. (B) The natural human bone with dual‐gradient porosity (smaller pores with thicker walls and larger pores with thinner walls) demonstrates negative Poisson's ratio structure, showing internal contraction under axial compression. (C) The RCS with negative Poisson's ratio structure also demonstrates internal contraction characteristics under axial compression. (D) The two‐dimensional negative Poisson's ratio structure of RCS transforms from planar to three‐dimensional configuration, ultimately forming a scaffold through stacking of the three‐dimensional structures.

A systematic approach was established to fabricate negative Poisson's ratio microstructures for integration within patient‐specific 3D‐printed hemipelvic implants. Initially, a symmetrical RCS was conceptualized and modeled utilizing SolidWorks 2019 (Dassault Systèmes SolidWorks Corp., France). Finite element analysis and mathematical modeling validated the auxetic behavior of this two‐dimensional configuration [30]. Subsequently, the planar geometry underwent spatial transformation via axial rotation, incorporating supporting struts to achieve uniform mechanical response across all loading directions. This modification enhanced the structural integrity while minimizing stress singularities and fabrication constraints. In the final phase, the optimized auxetic unit cell served as the fundamental building block, replicated in a periodic three‐dimensional lattice formation. The resultant architecture was strategically incorporated into the prosthetic design through Boolean algorithmic operations, yielding a biomechanically optimized implant featuring the novel chiral microstructure throughout its porous domains. The mechanical properties of the RCS design were validated through comprehensive finite element analysis using ANSYS Workbench 2020 (ANSYS Inc., Canonsburg, PA). Material properties were assigned based on Ti_6_Al_4_V characteristics (Young's modulus: 110 GPa, Poisson's ratio: 0.32, yield strength: 880 MPa). The RCS unit cells demonstrated an effective elastic modulus of 3–7 GPa under compressive loading, closely matching cortical bone properties (4–20 GPa). These mechanical characteristics are consistent with published computational studies demonstrating the auxetic behavior and enhanced fatigue resistance of RCS structures under cyclic loading conditions [31, 32] (Figure 1).

### 2.2 Integration of Auxetic Architecture in Patient‐Specific 3D‐Printed Pelvic Implants

#### 2.2.1 Comprehensive Prosthesis Design Protocol

**FIGURE 2:**
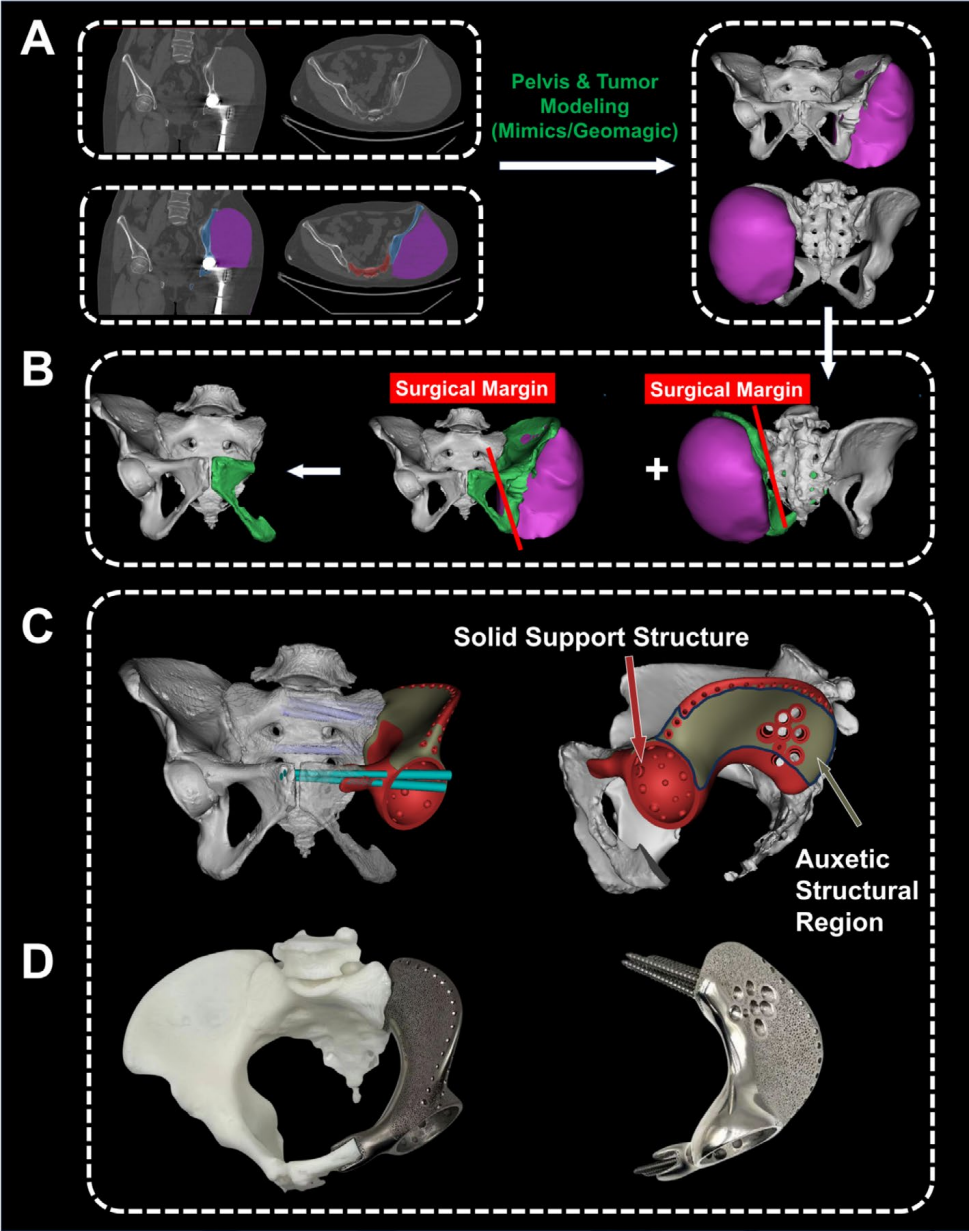
Design workflow of a custom 3D‐printed hemipelvic prosthesis. (A) Three‐dimensional reconstruction of the pelvis and tumor using Mimics software from preoperative CT data of a patient with recurrent high‐grade sarcoma (FNCLCC Grade 3). The tumor is shown in purple and the pelvis in white. (B) Computer‐simulated osteotomy planning with surgical margins (red lines) demonstrating the resultant pelvic ring defect. (C) Custom 3D‐printed prosthesis design for pelvic ring reconstruction, featuring a solid support structure and an auxetic structural region optimized for mechanical strength and functionality. (D) Physical prototype of the fabricated prosthesis.

The customized endoprostheses were manufactured by Chunli Co. Ltd. (Tongzhou, Beijing, China) based on designs developed by our surgical team. Our established protocol for prosthetic development, previously validated in clinical practice, encompassed multiple phases: (i) Advanced imaging fusion utilizing Three‐Dimensional Multimodality Image Reconstruction (TDMII) technology to synthesize CT and MRI datasets, generating high‐fidelity 3D pelvic‐tumor models for surgical planning [33]. (ii) Precise delineation of resection margins guided by tumor infiltration patterns and histopathological findings, with careful consideration for preserving critical anatomical structures, including the acetabulum, sacroiliac joint, and pubic symphysis. (iii) Detailed prosthetic engineering incorporating specific parameters for porous architecture, fixation strategy, optimal screw trajectories, and component positioning, all configured to accommodate the planned surgical approach (Figure 2).

#### 2.2.2 Implementation of re‐Entrant Chiral Auxetic Architecture

**FIGURE 3:**
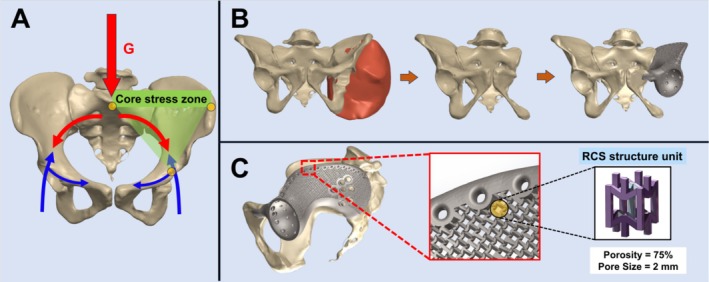
Strategic integration of RCS structure and mechanical adaptation of hemipelvic prosthesis. (A) Force transmission pattern in pelvis. Red arrow (G) indicates the primary body weight load, and the green zone highlights the core stress‐conducting area of pelvic ring where RCS structure is strategically integrated for optimal mechanical adaptation. Blue arrows demonstrate the reactive forces from the acetabulum. (B) Sequential illustration of tumor resection planning and prosthetic reconstruction. From left to right: Tumor extent (red), planned resection, and final prosthetic design. (C) Detailed view of the RCS‐enhanced prosthesis design. Magnified image shows the integration of porous structure with RCS unit cell specifications (porosity = 75%, pore size = 2 mm).

The novel RCS unit cell design was digitally integrated into the patient‐specific prosthesis using Materialize Magics CAD software (Materialize, Leuven, Belgium). To maximize biological and mechanical performance, the auxetic architecture was specifically distributed in two critical zones: the central prosthetic region for optimal load transfer and weight reduction, and the bone–implant interface to enhance osseointegration. A series of Boolean operations enabled precise control over the RCS array distribution, ensuring uniform porosity patterns with parameters optimized for both structural and biological requirements. The final prosthetic design, featuring the integrated auxetic framework, was converted to STL format for manufacturing using selective laser melting (SLM) technology with medical‐grade titanium alloy. Subsequently, nano‐hydroxyapatite (nHA) coatings were electrochemically deposited onto the RCS‐structured surfaces to provide dual osteogenic‐antitumor functionality [34, 35, 36, 37, 38, 39] (Figure 3).

### 2.3 Clinical Cohort Characteristics

Between March 2018 and June 2023, 15 consecutive patients underwent pelvic tumor resection and reconstruction utilizing RCS‐enhanced 3D‐printed hemipelvic prostheses. Patient selection followed strict inclusion criteria: histologically confirmed primary pelvic malignancy, technical feasibility for limb salvage surgery, and complete follow‐up documentation. Exclusion criteria included systemic contraindications, active infections, metal hypersensitivity, skeletal abnormalities, and incomplete follow‐up data.

The cohort comprised eight females and seven males, with a mean age of 39.3 ± 11.7 years (range: 19–65 years). Pathological diagnosis confirmed osteosarcoma in eight cases (53.3%), Ewing sarcoma in three cases (20%), chondrosarcoma in two cases (13.3%), and high‐grade soft tissue sarcoma in two cases (13.3%). All patients were staged as IIB according to the Enneking system, except for one Ewing sarcoma case (stage III) [40]. Neoadjuvant chemotherapy (four cycles) was administered to six osteosarcoma patients, and one Ewing sarcoma patient received six cycles of chemotherapy. The mean follow‐up duration was 44.5 ± 9.4 months (range: 36–62 months). Preoperative evaluation included comprehensive pathological assessment, staging studies, and multimodal imaging (radiography, CT, MRI, and SPECT). Detailed patient demographics and clinical characteristics are presented in Table 1. The study protocol was approved by our institutional review board, and informed consent was obtained from all participants.

**TABLE 1:** Demographic and clinical characteristics of patients undergoing RCS‐enhanced 3D‐printed hemipelvic reconstruction.

No.	BMI (kg/m^2^)	Diagnosis	Stage	Tumor location	Preop chemo	Op time (min)	Blood loss (mL)	Pre‐MSTS	Post‐MSTS	Pre‐VAS	Post‐VAS	Follow‐up (months)	Outcome
1	24.3	Osteosarcoma	IIB	II + III	NA	265	3200	14	26	6	1	46	Disease‐free
2	23.1	Osteosarcoma	IIB	II + III	4 cycles	310	4100	15	27	5	2	52	Disease‐free
3	25.6	Chondrosarcoma	IIB	I + II	NA	245	2800	16	28	5	1	38	Disease‐free
4	22.4	Osteosarcoma	IIB	I + II + III	4 cycles	330	4500	13	25	6	2	32^a^	Died of metastasis
5	24.8	Osteosarcoma	IIB	II + III	NA	280	3300	15	26	5	1	42	Disease‐free
6	23.7	Osteosarcoma	IIB	I + II + III + IV	4 cycles	295	3800	14	24	6	2	58	Disease‐free
7	25.2	Osteosarcoma	IIB	II + III	NA	255	2900	16	27	5	1	44	Disease‐free
8	21.8	Ewing sarcoma	III	I + II + III + IV	6 cycles	360	4800	12	23	7	2	62	Disease‐free
9	24.5	High‐grade soft tissue sarcoma	IIB	II + III	NA	270	3100	15	26	5	1	40	Disease‐free
10	23.9	Osteosarcoma	IIB	I + II + III	4 cycles	315	4200	14	25	6	2	54	Disease‐free
11	24.1	Ewing sarcoma	IIB	I + II	NA	275	3000	15	27	5	1	36	Disease‐free
12	22.8	Osteosarcoma	IIB	I + II + III	4 cycles	305	4000	14	26	6	2	28^b^	Local recurrence
13	25	Chondrosarcoma	IIB	II + III	NA	260	3200	15	26	5	1	42	Disease‐free
14	24.4	Ewing sarcoma	IIB	II + III	NA	285	3400	15	25	5	1	45	Deep infection^c^
15	23.2	High‐grade soft tissue sarcoma	IIB	II + III	NA	290	3600	14	26	6	2	48	Disease‐free
Statistical analysis								Mean ± SD	14.5 ± 1.1	25.8 ± 1.3	5.5 ± 0.6	1.5 ± 0.5	44.5 ± 9.4
*p* value (paired *t* test)										< 0.001		< 0.001	

Abbreviations: BMI, body mass index; MSTS, Musculoskeletal Tumor Society functional evaluation; NA, not applicable; Op Time, operation time; SD, standard deviation; VAS, Visual Analog Scale.

^a^ Patient died of metastatic complications at 32 months post‐surgery.

^b^ Patient experienced local recurrence at 28 months post‐surgery and underwent revision surgery.

^c^ Patient developed deep prosthetic infection at 14 months post‐surgery; managed with debridement and antibiotics.

### 2.4 Surgical Procedures and Postoperative Management

All surgical procedures were performed following precise computer‐assisted preoperative simulation. The surgical approach was tailored based on tumor location and planned resection margins, with patient‐specific cutting guides applied. These cutting guides were designed through a systematic process: high‐resolution CT imaging was used for 3D pelvic reconstruction, followed by virtual tumor resection planning with oncologically safe margins. Computer‐aided design software was then employed to create cutting templates that precisely matched the planned osteotomy planes, ensuring accurate bone cuts that align perfectly with the prosthetic interface geometry. The guides were 3D‐printed using biocompatible materials and sterilized prior to surgery, minimizing intraoperative adjustments and enhancing surgical precision. This approach facilitated accurate osteotomy using an ultrasonic bone scalpel.

Prosthetic fixation technique varied according to anatomical involvement and resection pattern. In periacetabular resections, initial fixation began proximally at the sacral interface, where the RCS‐enhanced porous surface was intimately positioned against prepared trabecular bone, followed by strategic screw placement along pre‐planned trajectories. For pubic reconstruction, a custom porous‐surfaced stem was press‐fitted into the remaining bone stock, while additional screws secured the ischial region when preserved. The acetabular component was cemented with 5°–10° anteversion to optimize stability while minimizing dislocation risk. Meticulous attention was paid to soft tissue reconstruction, particularly the hip abductors and capsular repair, to enhance joint stability.

Postoperatively, the operated limb was positioned to optimize healing and minimize mechanical stress (15°–25° abduction, 15° flexion at both hip and knee joints, neutral rotation). A standardized rehabilitation program, developed through institutional experience [41, 42], was implemented. Systematic follow‐up was scheduled monthly for 3 months, quarterly for 2 years, and biannually thereafter. Assessment included oncological surveillance, functional evaluation (MSTS‐93), surgical outcome analysis, pain assessment (VAS), complication monitoring, and osseointegration evaluation using T‐SMART technology.

### 2.5 Statistical Analysis

Data analysis was conducted using SPSS 21.0 (IBM, Armonk, NY). Variables were tested for normality using the Shapiro–Wilk test. Normally distributed data (operative time, blood loss, MSTS‐93, and VAS scores) were analyzed using paired t‐tests, while nonparametric data underwent Mann–Whitney *U* testing. For all analyses, statistical significance was set at *p* < 0.05 (two‐tailed).

## 3 Results

### 3.1 Oncological Outcomes and Functional Assessments

At the final follow‐up evaluation (44.5 ± 9.4 months), 13 patients (86.7%) remained disease‐free, one patient (6.7%) presented with local recurrence at 28 months post‐surgery, and one patient (6.7%) succumbed to metastatic complications at 32 months post‐surgery. The patient with local recurrence underwent revision surgery with wider margins and adjuvant radiotherapy. Functional assessment demonstrated significant improvement across all patients. The mean MSTS‐93 score increased significantly from 14.5 ± 1.1 points (range: 12–16) preoperatively to 25.8 ± 1.3 points (range: 23–28) at the final follow‐up (*p* < 0.001). Analysis of individual MSTS domains revealed the most significant improvements in: pain relief (2.1 ± 0.3 to 4.8 ± 0.4, *p* < 0.001), functional activities (2.3 ± 0.4 to 4.5 ± 0.5, *p* < 0.001), and emotional acceptance (2.0 ± 0.4 to 4.3 ± 0.4, *p* < 0.001). Walking ability and gait also showed substantial improvement (2.2 ± 0.3 to 4.2 ± 0.4, *p* < 0.001). All surviving patients achieved satisfactory functional recovery, demonstrating the ability to perform daily activities including cross‐legged sitting and squatting, with adequate weight‐bearing capacity.

### 3.2 Surgical Outcomes and Pain Control Assessment

The implementation of computer‐assisted navigation and patient‐specific cutting guides facilitated precise surgical execution. En bloc resection was achieved in all cases, with negative margins confirmed by postoperative histopathological examination. The mean operative time was 289.3 ± 30.4 min (range: 245–360 min), with an average intraoperative blood loss of 3540 ± 621.5 mL (range: 2800–4800 mL). Pain management outcomes showed marked improvement, with mean VAS scores decreasing from 5.5 ± 0.6 points (range: 5–7) preoperatively to 1.5 ± 0.5 points (range: 1–2) at final follow‐up. All patients demonstrated progressive improvement in pain scores during the rehabilitation period.

### 3.3 Complications

Early postoperative complications included delayed wound healing in three patients (20%), successfully managed through wound care protocols and VAC therapy. One patient (6.7%) developed a deep prosthetic infection at 14 months post‐surgery. Treatment involved a two‐stage revision procedure: initial implant removal with thorough debridement, placement of an antibiotic‐loaded cement spacer, and administration of systemic antibiotics for 12 weeks, followed by reimplantation of a new RCS‐enhanced prosthesis after confirmation of infection eradication. No cases of prosthetic loosening, mechanical failure, dislocation, or periprosthetic fracture were observed throughout the follow‐up period. The overall complication rate (26.7%) was comparable to previously reported rates for pelvic reconstructions, suggesting that the RCS‐enhanced design did not introduce additional complications compared to conventional prostheses.

### 3.4 Radiographic Outcomes

**FIGURE 4:**
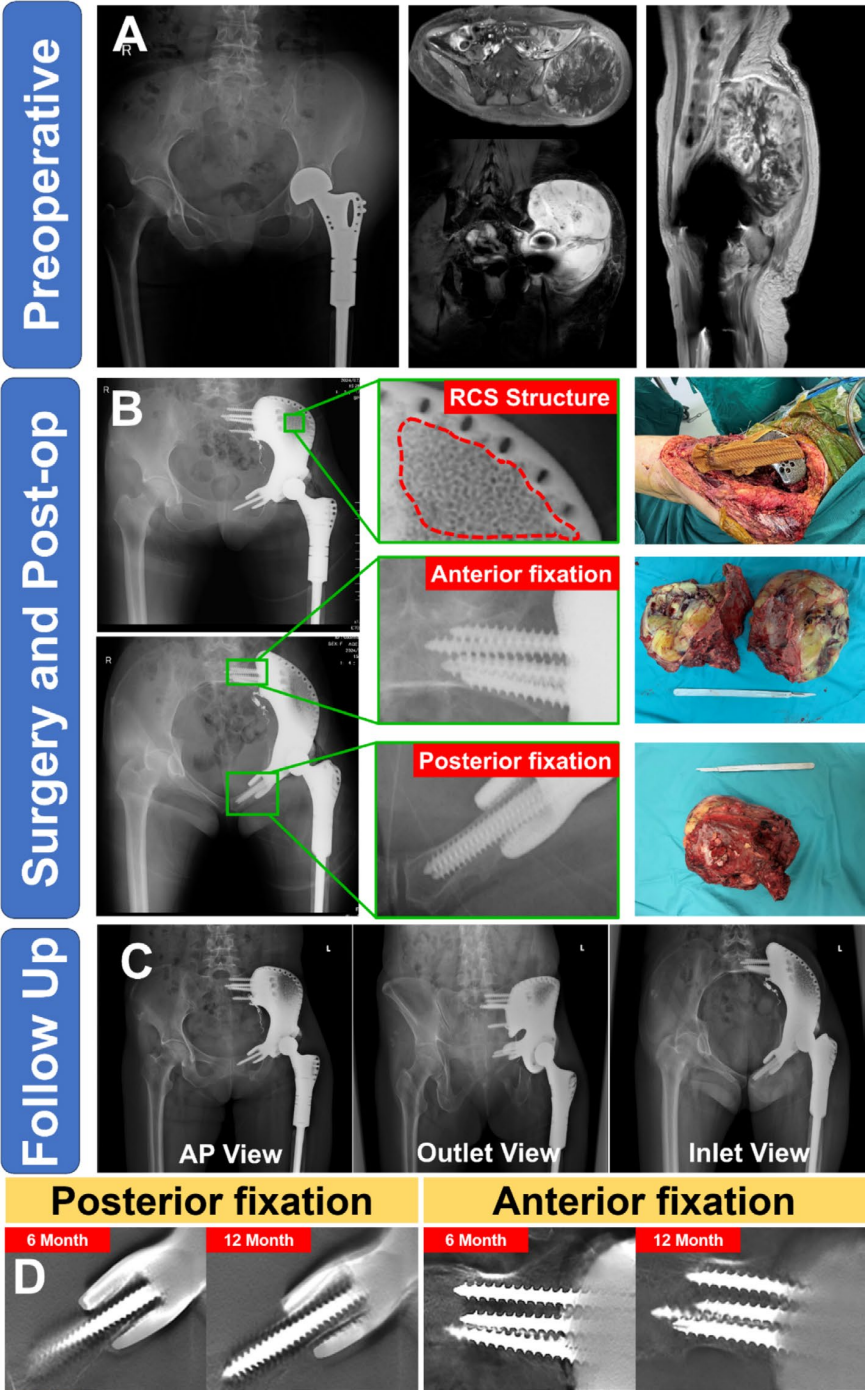
Clinical application of 3D‐printed custom hemipelvic prosthesis with Re‐entrant Chiral Structure (RCS). (A) Preoperative radiograph and magnetic resonance imaging of a patient with recurrent high‐grade sarcoma (FNCLCC Grade 3). Images demonstrate tumor infiltration of the pelvis and surrounding soft tissues following initial surgery. (B) Intraoperative resection specimen and postoperative radiographic assessment. Magnified views highlight the specialized RCS porous architecture (top), anterior fixation (middle), and posterior fixation (bottom). The prosthesis demonstrates accurate positioning with optimal bone–implant interface contact. (C) Immediate postoperative radiographs (AP, outlet, and inlet views) showing accurate prosthesis positioning and anatomical alignment. (D) Serial T‐SMART follow‐up imaging at 6 and 12 months demonstrating progressive osseointegration at both posterior and anterior fixation sites. The bone–prosthesis interface shows continued stability and enhanced integration over time, with no evidence of loosening or migration.

Postoperative imaging confirmed accurate prosthesis positioning and screw placement in all cases, consistent with preoperative planning parameters. Serial radiographic evaluation demonstrated progressive osseointegration at the bone‐prosthesis interface, particularly evident in the RCS‐enhanced porous regions. T‐SMART analysis revealed no signs of osteolysis or implant loosening at the final follow‐up. The clinical case presented in Figure 4 illustrates successful prosthetic integration and maintained alignment at 12 months post‐surgery.

## 4 Discussion

The comprehensive treatment of pelvic malignancies presents a dual challenge: achieving wide oncological resection and restoring stable biomechanical function. While various reconstructive approaches exist, the integration of advanced biomaterials and manufacturing techniques represents a significant step forward. Our study demonstrates that 3D‐printed hemipelvic prostheses incorporating a RCS offer a promising solution, achieving a unique synergy between mechanical integrity, biological integration, and favorable clinical outcomes.

### 4.1 Biomechanical Superiority of the RCS Design Translates to Clinical Durability

A fundamental innovation of our approach lies in the RCS architecture's ability to overcome the traditional trade‐off between porosity and strength in metallic implants. Conventional porous titanium implants are typically restricted to < 40% porosity to maintain structural integrity, falling short of the > 60% porosity considered optimal for osteoconduction [19, 20]. In contrast, our RCS design achieves a porosity of 75% while preserving mechanical robustness. This is accomplished through the material's auxetic properties, specifically its negative Poisson's ratio, which leads to more uniform stress distribution and enhanced resistance to localized stress concentrations under load [22, 43].

The clinical relevance of this biomechanical advantage is significant. The RCS structure's effective elastic modulus (3–7 GPa) closely matches that of cortical bone (4–20 GPa), which is crucial for mitigating stress shielding—a common failure mechanism in overly stiff conventional implants [21]. Furthermore, studies have shown that auxetic structures possess superior fatigue resistance under the complex, cyclic loading conditions experienced in the pelvis [44]. This enhanced durability is supported by our clinical findings, as no mechanical failures, aseptic loosening, or prosthesis fractures were observed in any patient during the follow‐up period, providing preliminary evidence of the design's in vivo mechanical integrity.

### 4.2 Favorable Mechanics and High Porosity Promote Effective Osseointegration

The biomechanical environment created by the RCS prosthesis appears to be highly conducive to bone ingrowth. Postoperative radiographic analysis using T‐SMART technology consistently demonstrated progressive osseointegration at the bone‐prosthesis interface, with no signs of osteolysis or implant migration. This successful biological fixation can be attributed to several factors.

At the macro level, the high porosity (> 60%) and interconnected pore networks (pore size = 2 mm) facilitate deep cellular infiltration and neovascularization, which are prerequisites for bone formation [45]. At the micro‐level, auxetic structures may enhance mechanotransduction signals to resident osteogenic cells [46]. The dynamic deformation of the RCS framework under physiological loading can create unique fluid flow patterns within the porous network, which is known to stimulate osteoblast activity and matrix production [47]. This combination of a stable, bone‐mimicking mechanical environment and an open porous architecture provides an ideal scaffold for durable biological fixation.

### 4.3 Structural and Biological Advantages Yield Favorable Clinical and Functional Outcomes

Ultimately, the success of any reconstructive technique is measured by its impact on the patient's quality of life. In our cohort, the structural and biological advantages of the RCS prosthesis translated into significant clinical benefits. The mean MSTS‐93 functional score improved dramatically from 14.5 preoperatively to 25.8 postoperatively (*p* < 0.001), indicating substantial functional recovery [40]. Similarly, pain control was excellent, with mean VAS scores decreasing from 5.5 to 1.5 (*p* < 0.001). All surviving patients achieved satisfactory functional recovery, enabling them to perform daily activities with adequate weight‐bearing capacity.

These outcomes are particularly noteworthy given the complexity of pelvic reconstruction, which can significantly impact psychosocial well‐being and gait [48, 49]. Even the patient who developed a deep infection achieved a satisfactory functional outcome after successful two‐stage revision, highlighting the resilience of the overall limb‐salvage strategy. These results suggest that the stable fixation and reduced stress‐shielding provided by the RCS design create a strong foundation for effective soft tissue reconstruction and aggressive postoperative rehabilitation, leading to superior functional restoration.

### 4.4 Complications, Limitations, and Future Directions

While the outcomes are promising, we acknowledge the associated complications. One patient (6.7%) developed a deep prosthetic infection; another (6.7%) experienced a local tumor recurrence. These oncological and infection rates are consistent with those reported in other large series of 3D‐printed pelvic reconstructions [50], suggesting that the novel RCS design does not introduce additional risks compared to conventional custom prostheses.

Our study has several limitations. The retrospective design, small cohort size (*n* = 15), and heterogeneous patient population are inherent constraints. Additionally, while our mean follow‐up of 44.5 months demonstrates excellent early‐to‐mid‐term results, longer‐term studies are required to fully assess the implant's in vivo behavior and long‐term durability. Looking forward, the integration of bioactive coatings onto the RCS framework could further enhance osseointegration and potentially provide antitumor properties. Advanced imaging techniques and machine learning algorithms may also allow for patient‐specific optimization of RCS parameters, heralding a new frontier in personalized orthopedic oncology.

## 5 Conclusion

The incorporation of RCS in 3D‐printed titanium alloy prostheses demonstrates promising potential in hemipelvic reconstruction following tumor resection. This auxetic architecture offers superior mechanical properties while maintaining high porosity, potentially optimizing the balance between structural stability and biological integration. While long‐term studies are warranted, initial clinical outcomes suggest that RCS‐enhanced prostheses may represent an advancement in addressing the biomechanical challenges of pelvic reconstruction.

## Author Contributions

Conceptualization, supervision, project administration, funding acquisition, review and editing: Li Min, Xiao Yang. Methodology, formal analysis, original draft preparation: Linyun Tan, Ye Li. Investigation, data curation: Linyun Tan, Ye Li, Xin Hu, Yitian Wang, Yi Luo, Yong Zhou, Chongqi Tu. Validation: Xiaolu Zhang, Xiaoyan Liu. All authors have read and agreed to the published version of the manuscript.

## Ethics Statement

This single‐center retrospective study was performed in accordance with the 1964 Helsinki Declaration and was approved by the Medical Ethics Committee of West China Hospital, Sichuan University.

## Consent

Written informed consent was obtained directly from all patients aged 18 and above. For minors below 18 years of age, written informed consent was obtained from their parents or legal guardians.

## Conflicts of Interest

The authors declare no conflicts of interest.

## Data Availability

The data that support the findings of this study are available on request from the corresponding author. The data are not publicly available due to privacy or ethical restrictions.
